# Performance of Two-Phase Designs for the Time-to-Event Outcome and a Case Study Assessing the Relapse Risk Associated With B-ALL Subtypes

**DOI:** 10.1200/CCI-24-00223

**Published:** 2025-05-02

**Authors:** Wenan Chen, Ti-Cheng Chang, Karen R. Rabin, Elizabeth A. Raetz, Meenakshi Devidas, Stephen P. Hunger, Nilsa C. Ramirez, Charles G. Mullighan, Mignon L. Loh, Gang Wu

**Affiliations:** ^1^ Department of Health Sciences Research, Mayo Clinic, Rochester, MN; ^2^ Center for Applied Bioinformatics, St Jude Children's Research Hospital, Memphis, TN; ^3^ Department of Pediatrics, Baylor College of Medicine, Houston, TX; ^4^ Department of Pediatrics, Perlmutter Cancer Center, NYU Langone Hospital, New York, NY; ^5^ Global Pediatric Medicine, St Jude Children's Research Hospital, Memphis, TN; ^6^ Department of Pediatrics and the Center for Childhood Cancer Research, Children's Hospital of Philadelphia, Perelman School of Medicine, University of Pennsylvania, Philadelphia, PA; ^7^ Biopathology Center and Department of Pathology and Laboratory Medicine, Nationwide Children's Hospital, Columbus, OH; ^8^ Department of Pathology, St Jude Children's Research Hospital, Memphis, TN; ^9^ Department of Pediatrics, Ben Towne Center for Childhood Cancer and Blood Disorders Research, Seattle Children's Hospital, Fred Hutch Cancer Center, University of Washington, Seattle, WA

## Abstract

**PURPOSE:**

To reduce costs in genomic studies of time-to-event phenotypes like survival, researchers often sequence a subset of samples from a larger cohort. This process usually involves two phases: first, collecting inexpensive variables from all samples, and second, selecting a subset for expensive measurements, for example, sequencing-based biomarkers. Common two-phase designs include nested case-control and case-cohort designs. Additional designs include sampling subjects based on follow-up time, like extreme case-control designs. Recently an optimal two-phase design using a maximum likelihood-based method was proposed, which could accommodate arbitrary sample selection in the second phase. However, direct comparisons of this optimal design with others in terms of power and computational cost is lacking.

**METHODS:**

This study performs a direct evaluation of typical two-phase designs, including Tao's optimal design, on type I error, power, effect size estimation, and computational time, using both simulated and real data sets.

**RESULTS:**

Results show that the optimal design had the highest power and accurate effect size estimation under the Cox regression model. Surprisingly, logistic regression achieved similar power with much lower computational cost than a more sophisticated method. The study further applied these methods to the MP2PRT study, reporting hazard ratios of cancer subtypes on relapse risk.

**CONCLUSION:**

Recommendations for selecting two-phase designs and analysis methods are regarding power, bias of estimated effect size, and computational time.

## INTRODUCTION

**FIG 1.**
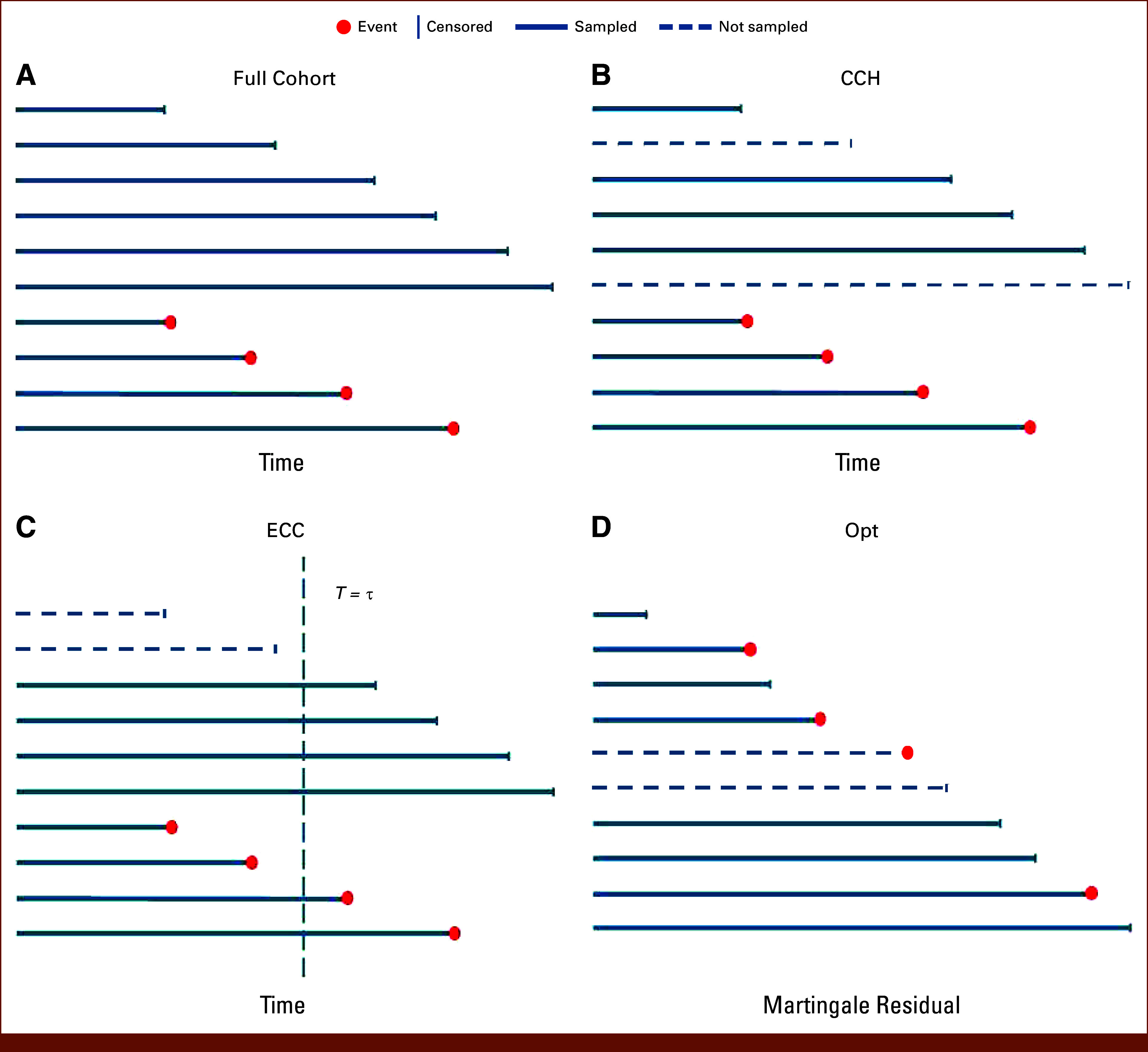
Study designs of time-to-event outcomes. Four different designs are illustrated assuming the same start time for all participants: (A) full cohort, (B) CCH, (C) ECC, and (D) Opt. The panel (D) shows the residual values instead of the follow-up time. Solid lines represent individuals sampled and dashed lines represent individuals not sampled in the study design. Note that for the stratified CCH and stratified ECC designs, subplot (B and C) can be viewed as illustrations from one stratum. CCH, case-cohort design; ECC, extreme case-control; Opt, optimal design.

When studying the effect of variables on the time-to-event outcome, a cohort is followed for a specific time period, and time-to-event information is collected. A typical design is the full cohort–based design (Fig 1A), where variables of interest from all individuals are measured. When measurements of variables are expensive to collect, the budget may only afford a subset of individuals. Therefore, subsampling of the full cohort in the second phase of collecting expensive variables is needed. There are two classical two-phase designs in time-to-event outcome analysis: nested case-control design (NCC)^1,2^ and case-cohort design (CCH).^3-5^ In general, these two designs are similar,^6^ so we consider the CCH (Fig 1B), and its variations such as the generalized CCH^7,8^ and the stratified CCH (SCCH).^9^ Another extension, which motivated this study, is a design that places an additional constraint for the selection of controls (Fig 1C), called extreme case-control (ECC) design because it shares similarity with previously proposed extreme case-control studies.^10,11^ It is not clear whether the ECC design is as efficient as other two-phase designs, although a recent study has shown that sampling extreme controls may have increased statistical power over NCC.^11^ Recently, Tao et al^12^ have proposed an optimal two-phase design for the time-to-event outcome. The corresponding maximum likelihood method has the advantage of dealing with arbitrary sample selection in the second phase. Therefore, in theory, it can also be applied to ECC design. However, in the study by Tao et al., there is no direct comparison with other two-phase designs in terms of statistical power and computational cost, which are important factors for analysis design of genome-wide analysis of sequencing data. In this study, we first evaluated the performance of important two-phase designs, and then applied different analysis methods to a real ECC design–based study.

**CONTEXT**

**Key Objective**
What cost-effective study designs and analysis methods should we choose when studying the association between expensive biomarkers and cancer treatment outcome?
**Knowledge Generated**
Recently proposed optimal design (Opt) shows the highest-statistical-power, logistic regression–based analysis is both powerful and scalable; for rare outcomes, stratified case-cohort design has small power loss compared with the Opt but has the advantage of mature analysis methods and flexibility of analyzing other outcomes. Hazard ratios of different subtypes of B-ALL with respect to the common subtype ETV6::RUNX1 for relapse risk after treatment are reported.
**Relevance *(Z. Bakouny)***
As biomarker studies employ increasingly complex and expensive genomics and other correlative methods, it is increasingly important to rigorously design these studies and analyze the downstream results. W.C. et al compare the performance of a set of study design and analysis methods and provide recommendations for future biomarker studies.*
*Relevance section written by *JCO Clinical Cancer Informatics* Associate Editor Ziad Bakouny, MD, MSc.

## METHODS

### Study Designs for the Time-to-Event Outcome

We summarize nine different study design and analysis method pairs considered in this study (Table 1). The first phase includes all samples with the observed time t, the event status δ, and inexpensive covariates measured for all samples. The second phase includes a subset of the samples to obtain the expensive covariates. Cases are those with observed event (δ=1) and controls are those without the event/censored (δ=0).

**TABLE 1.** Study Designs and Associated Analysis Methods Evaluated

Design	Case-Control Definition	Analysis Methods	Data Used in Analysis
Full cohort (cohort)	Case: δ = 1 Control: δ = 0	Cox	Cox: 1 + 2 (all)
Classical and generalized CCH	Case: δ = 1, sampled with proportion of p from all cases (p=1) for the classic case-cohort study Control: δ = 0, sampled with proportion of π from all controls	$\text{CoxW Weight}=\left\{ \begin{array}{c} \frac{1}{p}\;\text{case}\\ \frac{1}{\pi }\;\text{control} \end{array}\right.$	CoxW: 2 (subset)
SCCH	Case: δ = 1, sampled with proportion of $p_{i}$ from stratum iControl: δ = 0, sampled with proportion of $\pi_{i}$ from stratum i	$\text{CoxW Weight}=\left\{ \begin{array}{c} \frac{1}{p_{i}}\;\text{case}\;\text{in}\;\text{stratum}\;i\\ \frac{1}{\pi_{i}}\;\text{control}\;\text{in}\;\text{stratum}\;i \end{array}\right.$	CoxW: 2 (subset)
SECC	Case: δ = 1, sampled with proportion of $p_{i}$ from stratum iControl: δ = 0, sampled with a proportion $\pi_{i}$ from all controls satisfying the time constraint (*t* > τ) from stratum i	LG TaoLG TaoCox	LG: 2 (subset) TaoLG, TaoCox: 1 (all) + 2 (subset)
Opt	For each stratum i, sample $n_{i}$ cases and $n_{i}$ controls from the extreme martingale residuals, with optimized $n_{i}$	LG TaoLG TaoCox	LG: 2 (subset) TaoLG, TaoCox: 1 (all) + 2 (subset)

NOTE. δ indicates whether the event is observed or not. 1 + 2 (all): phase I and phase II of all samples; 2 (subset): phase II of the same subset of samples; 1 (all) + 2 (subset): phase I of all samples + phase II of a subset of samples.

Abbreviations: CCH, case-cohort design; Cox, Cox regression; CoxW, weighted Cox regression; LG, logistic regression; Opt, optimal design; SCCH, stratified case-cohort; SECC, stratified extreme case-control; TaoCox, maximum likelihood method from Tao et al^12^ using a Cox regression model; TaoLG, maximum likelihood method from Tao et al^12^ using a logistic regression model.

For the full cohort design (cohort; Fig 1A; Table 1), all samples are selected for the second phase, which is ideal but often infeasible because of the high cost. For the CCH (Fig 1B), all cases and a random subcohort is sampled in the second phase. CCH can be generalized where only a subset of cases are sampled, for example, there may be more cases than budget can afford or not all cases can be measured because of availability or quality issues. SCCH^9^ extends CCH by sampling cases or controls within strata defined by inexpensive covariates from the first phase, which can improve the statistical power of expensive covariates. The extreme case-control (ECC) design (Fig 1C) is similar to CCH, with the only difference being that the follow-up time of controls sampled in the second is above a threshold. Similarly, ECC can be extended to stratified ECC (SECC) by sampling within strata defined by inexpensive covariates. The optimal design (Opt; Fig 1D) recently proposed by Tao et al^12^ aims to minimize the variance of the estimated effects by an optima design, which uses information from both the second phase and the first phase. We followed the procedure proposed by Tao et al^12^ to calculate the martingale residuals and use the grid search to find the optimal number of individuals in each stratum defined by inexpensive covariates (Data Supplement).

### Analysis Methods

It is important for statistical models and analysis methods to reflect the essence of the study designs to be sound and powerful. Table 1 lists corresponding analysis methods and the input data for each design. For classical study designs, such as the full cohort design, CCH, and SCCH, we apply the mature methods Cox regression (Cox) or weighted Cox regression (CoxW). For recent study designs, such as SECC and the Opt, we apply three analysis methods: logistic regression (LG), Tao's maximum likelihood method using the logistic regression model (TaoLG), and Tao's maximum likelihood method using the Cox regression model (TaoCox). We note that the parameter of the effect size from the LG does not estimate the log hazard ratio directly. We include the method to evaluate its type I error under the null and power under the alternative hypothesis. Given that under rare disease assumption, odds ratio may approximate hazard ratio,^13^ we are interested in the extent of difference between the estimated log odds ratio and the log hazard ratio under different case proportions and the magnitude of effect sizes. More details are provided in the Data Supplement.

### Simulation Studies

We used a proportional hazard model to model covariate effects and Weibull distribution for the baseline hazard function using R package simsurv (v1.0.0).^14^ Specifically, the hazard function h(t) and the survival function S(t) are

$$h\left(t\right)=\gamma \lambda \left(t^{\gamma -1}\right)\exp\left(z^{\prime }\beta \right)$$ (1)

$$S\left(t\right)=\exp\left(-\lambda \left(t^{\gamma }\right)\exp\left(z^{\prime }\beta \right)\right)$$ (2)

where γ and λ are the shape and scale parameters of the Weibull distribution, respectively. z is the covariate vector and β is the covariate effect, that is, log hazard ratio. We considered one inexpensive covariate and one expensive covariate in the model with two scenarios between them: independent and correlated. Different parameter settings were simulated (Data Supplement, Table S1). We first simulated the full cohort of sample size 3,000, and sampled 100 cases and 100 controls for other designs. There were 2,000 replicates for each setting for evaluation.

Besides type I error and power, we also compared the estimation of the log hazard ratio using two metrics: the relative bias (bias divided by the true value) and the relative root mean square error (RMSE) defined as the ratio between RMSE of each method and RMSE of the CCH (Data Supplement).

### Real Data Analysis

The MP2PRT Study^15^ profiled B-ALL patients with multiomics data including whole-genome sequencing (WGS), whole-exome sequencing, and RNA-seq, to study the association between the molecular profiles at diagnosis and treatment outcomes. One important treatment outcome was the relapse status after treatment. The patients with B-ALL were collected from four clinical trials between 2003 and 2019 comprising two trials of standard risk and two trials of high risk but with favorable cytogenetic features. The proportion of relapse was about 10% for patients with B-ALL.^16^ We considered the two standard-risk cohorts AALL0331^16^ and AALL0932^17^ here, which comprised the majority of the profiled samples in the MP2PRT study. Both AALL0331 and AALL0932 are randomized prospective clinical trials of patients with standard-risk B-ALL designed to compare different treatment options, which do not show significant differences on the basis of later analyses.^16,17^ The recruitment of the phase I data does not depend on the relapse status, which is a later event. Minimal residual disease (MRD) at the end of induction was known to be positively associated with the risk of relapse, and positive MRD (MRD >0.01%) was matched among the cases and controls within each trial, to identify additional risk factors beyond MRD in the second phase. The expensive variable of interest was the subtype of B-ALL on the basis of multiomics profiling. Other expensive variables that were only available in the case-control cohort were age, sex, and race. The MRD status and clinical trial identifier were available for all patients in the two trials. We applied LG, TaoLG, and TaoCox in the analysis.

### Ethical Considerations

The clinical trials included here were approved by the Pediatric Central Institutional Review Board (IRB) of the National Institutes of Health, and participating institutional IRBs. Written informed consent and assent (if applicable) were obtained before study entry for each of the trials.

## RESULTS

### Type I Error and Power

**FIG 2.**
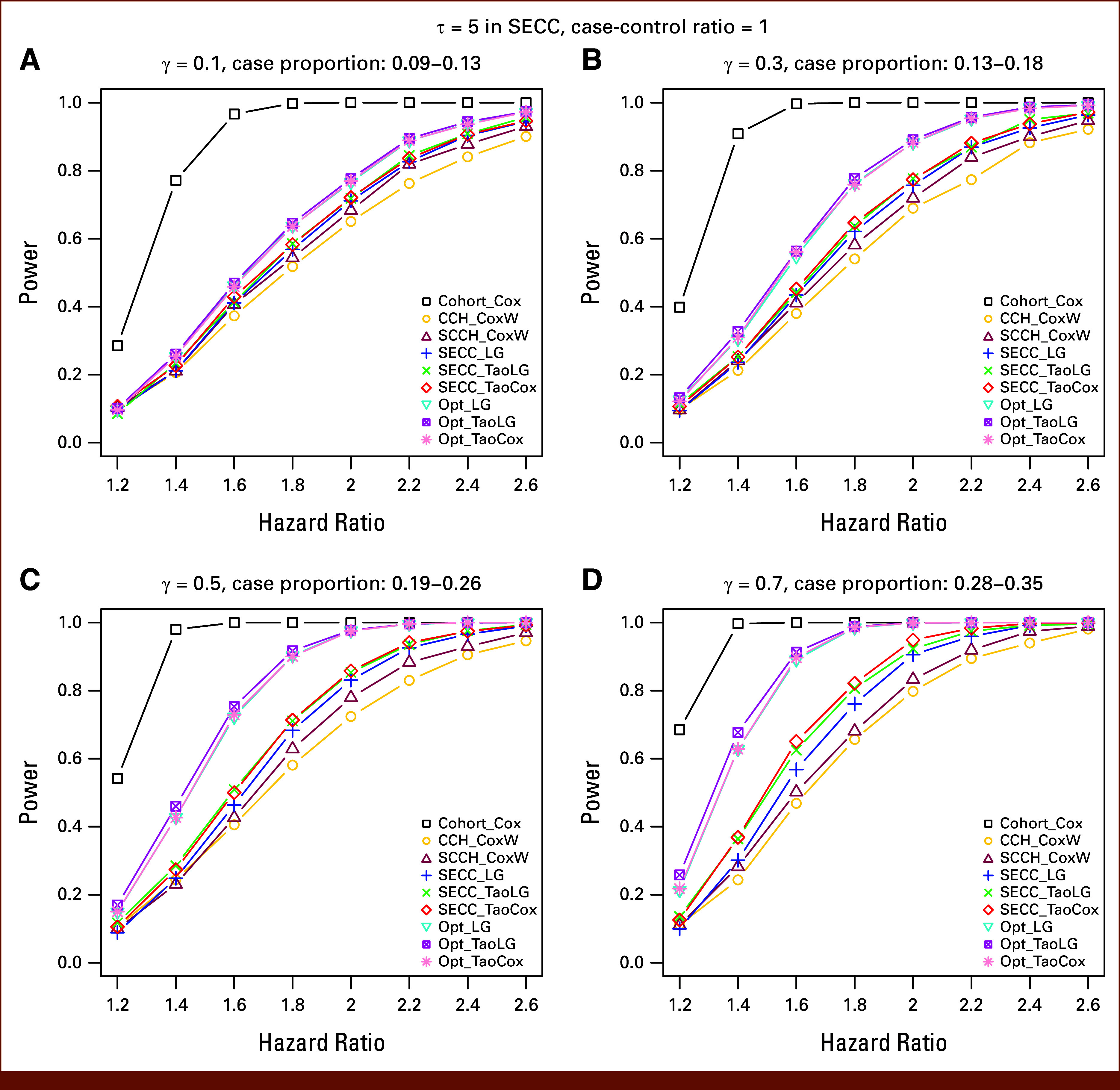
Empirical power of different designs and analysis methods: (A) γ = 0.1, case proportion: 0.09−0.13, (B) γ = 0.3, case proportion: 0.13−0.18, (C) γ = 0.5, case proportion: 0.19−0.26, and (D) γ = 0.7, case proportion: 0.28−0.35. CCH, case-cohort design; Cox, Cox regression; CoxW, weighted Cox regression; LG, logistic regression; Opt, optimal design; SCCH, stratified case-cohort; SECC, stratified extreme case-control; TaoCox, Tao's maximum likelihood method using the Cox regression model; TaoLG, Tao's maximum likelihood method using the logistic regression model.

We focused on the results where the specified expensive covariate was correlated with the inexpensive covariate. The setting where the expensive covariate is independent from the inexpensive covariate shows similar patterns. First, type I error for all designs and methods are well controlled at the nominal level 0.05 (Data Supplement, Figs S1 and S4). The statistical powers of three methods applied to the Opt (Opt_TaoCox, Opt_TaoLG, and Opt_LG) are remarkably similar, which are higher than other two-phase designs (Fig 2; Data Supplement, Fig S5). The power advantage of the Opt is larger when the case proportion increases. When the event is rare, for example, <0.13, the difference in power between the Opt and the stratified design is small. The power of stratified design (SCCH and SECC) is slightly higher than the CCH without stratification (CCH). SECC shows similar power as SCCH when the case proportion is rare, for example, <0.13 (Fig 2A), and slightly higher when the case proportion becomes larger, for example, >0.19 (Figs 2C and 2D). Tao's maximum likelihood–based method (SECC_TaoLG and SECC_TaoCox) shows similar or slightly better power than LG (SECC_LG) for the SECC.

### Bias and RMSE

For methods estimating the hazard ratio, there are no obvious biases, including TaoCox for the extreme case-control design and the Opt (Data Supplement, Figs S2 and S6). For methods estimating the odds ratio, the bias depends on the design, case proportion, and effect size. When the case proportion is small (<0.13), the relative bias of SECC_LG is <0.2, with higher bias from Opt_LG. The bias increases as the magnitude of effect sizes increases and the case proportion increases (Data Supplement, Figs S2C and S2D and S6C and S6D), consistent with previous theoretical analysis.^13^

For RMSE (Data Supplement, Figs S3 and S7), when the case proportion is small, for example, <0.13, and the effect sizes are small, for example, <2, all two-phase designs show similar results except slightly higher RMSE for SECC_LG and Opt_LG, and slightly lower RMSE of Opt_TaoCox. For methods estimating the hazard ratio, the relative RMSE in general reflects the power of different designs: the lower the relative RMSE, the higher the power. For large case proportion, the Opt with Opt_TaoCox shows a clear advantage in RMSE than other two-phase designs. SECC_TaoCox shows slightly smaller RMSE than CCHs. For methods estimating the odds ratio, the relative RMSE increases as the magnitude of the effect size and the case proportion increase. TaoLG shows similar general patterns as LG for bias and RMSE.

### Computational Cost

We reported the average computing time over different γ values and replicates when the inexpensive and expensive covariates are corelated (Data Supplement, Table S2), similar results were observed for independent covariates. LG had approximately 100-fold speedup over TaoLG, and approximately 2,000-fold speedup over TaoCox.

### Summary of Simulation Results and Recommendations to Sequencing Designs for Studying Relapse Risk

Advantages and limitations of different designs are summarized in Table 2. In general, when the case proportion is small, for example, <0.13, the differences in power among all two-phase designs are small. If there is enough budget, the full cohort is the best option, being the most powerful and flexible. Otherwise, the two-phase designs should be considered. Mature designs include NCC, CCH, and SCCH with mature analysis tools. Furthermore, because CCH and SCCH use the weighted method to account for the sampling in the design, it is straightforward to analyze other outcomes. For rare events, for example, case proportion <10%, although the Opt can have slightly higher power, a more sophisticated analysis method is needed to estimate the hazard ratio. The computational cost is much higher with the current implementation. The power advantage of Opt may disappear for other outcomes. Therefore, when multiple outcomes are of interest, a SCCH is likely a better choice. For common events, for example, case proportion >20%, Opt demonstrates a large power advantage. When SECC or Opt is adopted, if we are only interested in identifying significant associations, LG and TaoLG are good options because there is almost negligible power loss compared with TaoCox, but with much better scalability. If the number of biomarkers to test is small, TaoCox can be applied for estimating the hazard ratios.

**TABLE 2.** Advantages and Limitations of Different Study Designs

Design	Advantages	Limitations	Analysis Methods
Full cohort (cohort)	Mature design and analysis Can estimate hazard ratios Can analyze >1 outcomes	Not cost-effective when the sample size is large	Cox regression
Classical and generalized CCH	Mature design and analysis Cost-effective Can estimate hazard ratios Can analyze >1 outcomes	Large power loss compared with Opt when the case proportion is high	CoxW
SCCH	Mature design and analysis Cost-effective Can estimate hazard ratios Can analyze >1 outcomes Improved power over CCH	Large power loss compared with Opt when the case proportion is high	CoxW
SECC	Cost-effective Can estimate hazard ratios Improved power over SCCH	Large power loss compared with Opt when the case proportion is high LG-based analysis is fast but only provides odds ratios, not the hazard ratios. TaoCox is slow, but provides estimated hazard ratios Analyzing other outcomes is not trivial	LG TaoLG TaoCox
Opt	The most powerful and cost-effective design for one outcome Large power gain when the case proportion is high Can estimate hazard ratios	LG-based analysis is fast but only provides odds ratios, not the hazard ratios. TaoCox is slow, but provides estimated hazard ratios The power advantage is specific to one outcome Analyzing other outcomes is not trivial	LG TaoLG TaoCox

Abbreviations: CCH, case-cohort design; CoxW, weighted Cox regression; LG, logistic regression; Opt, optimal design; SCCH, stratified case-cohort; SECC, stratified extreme case-control; TaoCox, Tao's maximum likelihood method using the Cox regression model; TaoLG, Tao's maximum likelihood method using the logistic regression model.

### Application to MP2PRT Data

For MP2PRT, two covariates, MRD and clinical trial identifier, were available for the full cohort, and four covariates, cancer subtype, age at diagnosis, sex, and race, were only available for the selected case-control cohort (Data Supplement, Table S3). We applied LG, TaoLG, and TaoCox to study the association between covariates, mainly B-ALL subtype, and the relapse status. Subtype *ETV6::RUNX1* was set as the reference subtype. Subtypes with less than 10 samples were merged into one single category in the analysis. We also merged the Pacific Islander group (n = 2) with the Native American group (n = 6). Because there was matching on MRD status for the SECC design, it was expected that we would not detect the association between MRD and relapse using LG on phase II data. However, TaoLG and TaoCox were expected to detect the association between MRD and the relapse using both phase I and phase II data.

**FIG 3.**
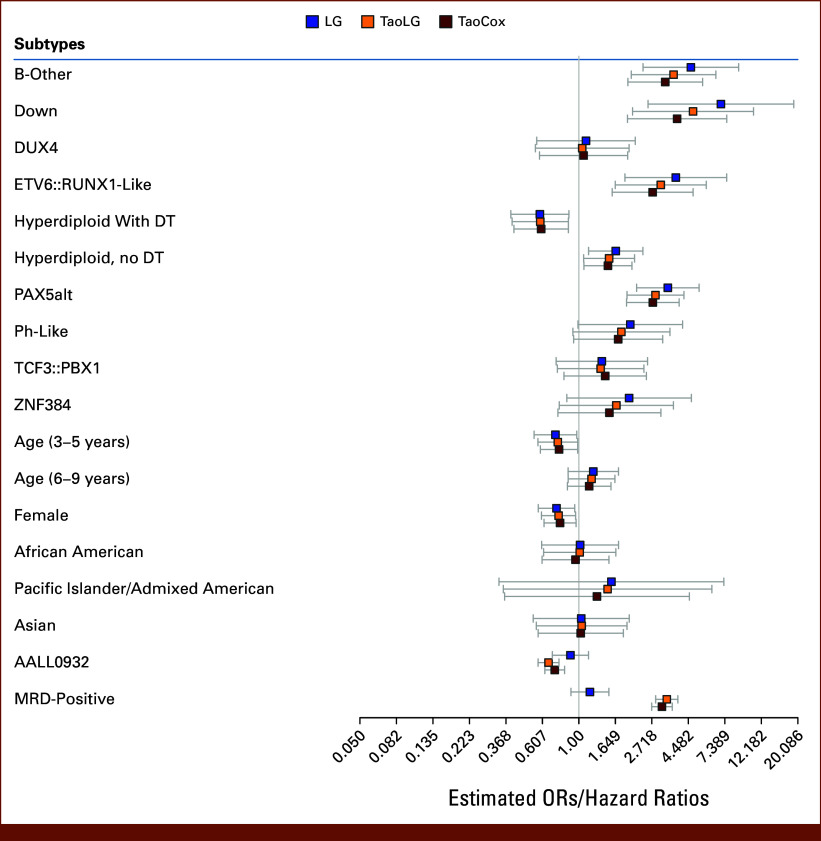
Estimated hazard ratios/ORs and 95% CIs for subtypes and other risk factors on relapse status. The squares on the lines are the estimated effects of different methods. The lines represent 95% CIs of the estimated effects. The *x*-axis is in log scale. DT, double trisomy; LG, logistic regression; MRD, minimal residual disease; ORs, odds ratios; TaoCox, Tao's maximum likelihood method using the Cox regression model; TaoLG, Tao's maximum likelihood method using the logistic regression model.

All methods identified the same set of cancer subtypes that had significantly different effect sizes from the reference subtype *ETV6::RUNX1*, with similar *P* values (Table 3). The same pattern was observed for other phase II covariates. This was consistent with our simulations showing similar power among LG, TaoLG, and TaoCox. The point estimate and the 95% CI were close in general when the effect size was small, for example, hyperdiploid with double trisomy (DT) and hyperdiploid without DT (Fig 3), consistent with the simulation results of estimated effect sizes. When the effect size was large, for example, Down syndrome, *ETV6::RUNX1*-like, and PAX5alt, the point estimates showed larger differences than those with smaller effect sizes. For those statistically significant subtypes, the bias of LG and TaoLG was toward increased magnitude of effect size (in log scale), consistent with simulation.

**TABLE 3.** *P* Values and Estimated ORs and Hazard Ratios of Risk Factors on Relapse Status

Subtypes	LG				TaoLG				TaoCox			
	OR	Lower	Upper	*P*	OR	Lower	Upper	*P*	HR	Lower	Upper	*P*
B-other	4.64	2.41	8.93	**4.48E-06**	3.66	2.05	6.53	**1.14E-05**	3.27	1.96	5.45	**5.94E-06**
Down	7.00	2.58	19.02	**1.34E-04**	4.78	2.09	10.95	**2.16E-04**	3.84	1.95	7.59	**1.05E-04**
DUX4	1.10	0.56	2.16	7.78E-01	1.05	0.55	1.99	8.88E-01	1.07	0.58	1.95	8.35E-01
ETV6::RUNX1-like	3.77	1.88	7.58	**1.89E-04**	3.07	1.65	5.73	**4.25E-04**	2.75	1.58	4.78	**3.55E-04**
Hyperdiploid with DT	0.59	0.39	0.87	**8.46E-03**	0.59	0.40	0.87	**7.12E-03**	0.60	0.41	0.87	**6.59E-03**
Hyperdiploid, no DT	1.66	1.14	2.40	**7.78E-03**	1.51	1.07	2.14	**1.98E-02**	1.49	1.07	2.07	**1.78E-02**
PAX5alt	3.38	2.20	5.19	**2.42E-08**	2.86	1.93	4.22	**1.36E-07**	2.76	1.92	3.95	**3.46E-08**
Ph-like	2.02	0.99	4.14	5.41E-02	1.79	0.92	3.48	8.59E-02	1.71	0.93	3.15	8.39E-02
TCF3::PBX1	1.37	0.73	2.56	3.25E-01	1.35	0.74	2.44	3.25E-01	1.43	0.82	2.52	2.11E-01
ZNF384	1.99	0.85	4.67	1.14E-01	1.67	0.76	3.65	1.98E-01	1.52	0.75	3.08	2.45E-01
Age (3-5 years)	0.73	0.54	0.97	**3.12E-02**	0.75	0.57	0.98	**3.79E-02**	0.76	0.59	0.98	**3.72E-02**
Age (6-9 years)	1.22	0.86	1.72	2.62E-01	1.19	0.86	1.64	2.88E-01	1.15	0.85	1.55	3.55E-01
Female	0.74	0.57	0.94	**1.60E-02**	0.76	0.60	0.96	**1.97E-02**	0.77	0.62	0.96	**2.11E-02**
African American	1.02	0.60	1.72	9.50E-01	1.01	0.62	1.66	9.63E-01	0.95	0.60	1.51	8.43E-01
Asian	1.03	0.53	1.99	9.24E-01	1.04	0.56	1.93	9.05E-01	1.03	0.57	1.84	9.33E-01
Native American^a^	1.56	0.33	7.29	5.71E-01	1.48	0.35	6.19	5.90E-01	1.28	0.36	4.54	7.01E-01
AALL0932	0.89	0.70	1.14	3.59E-01	0.66	0.57	0.76	**1.26E-08**	0.72	0.63	0.82	**1.65E-06**
MRD-positive	1.16	0.90	1.51	2.56E-01	3.33	2.86	3.88	**2.45E-54**	3.12	2.71	3.59	**1.15E-56**

NOTE. Values in bold indicate *P* < .05.

Abbreviations: DT, double trisomy; HR, hazard ratio; LG, logistic regression; Lower, Upper, the lower and upper bounds of the 95% CI; MRD, minimal residual disease; OR, odds ratio; TaoCox, Tao's maximum likelihood method using the Cox regression model; TaoLG, Tao's maximum likelihood method using the logistic regression model.

a This group is merged with another two Pacific Islander patients.

One important discovery of the MP2PRT Study was the high relapse risk of the recently defined subtype PAX5alt.^18^ However, the effect size was reported as the odds ratio from previous analysis using LG.^15^ Our analysis using TaoCox provided the estimated hazard ratio of PAX5alt, which was 2.76 compared with the reference subtype *ETV6::RUNX1* after adjusting for other covariates. Other subtypes with hazard ratios above two were *ETV6::RUNX1*-like, Down syndrome, and B-other. TaoCox also provided the estimated effect of the MRD status (hazard ratio = 3.12) for the full cohort, which could not be estimated using LG. Given the much larger number of samples in the phase I full cohort, the two clinical trials also showed significantly different relapse risk using TaoLG and TaoCox. In addition, the analysis showed that female patients had reduced relapse risk compared with male patients, and the diagnosed age (3-5 years) had reduced relapse risk than the reference age group with age <3 years.

For the computational cost, LG took about 0.02 seconds and TaoLG took about 266 seconds. For TaoCox, it took approximately 23 hours to finish, which would make it infeasible for a genome-wide scan between genetic markers and the outcome. The even higher cost from TaoLG and TaoCox in this analysis than those in simulations may also be related to the fact that the maximum likelihood methods primarily target a single association parameter, which may complicate its application when multiple expensive covariates are present.

## DISCUSSION

We evaluated four different two-phase designs for expensive covariates in the second phase, in the context of genomic sequencing studies for identifying the association between genomic markers and cancer relapse risk. Opt showed the highest power and consistent effect size estimation under the Cox model. We also showed that the power of LG and the more sophisticated maximum likelihood methods were similar for both Opt and the extreme case-control design. Given the fast computing time, LG can be an efficient method for identifying association for extreme case-control design or Opt when statistical power is the primary aim. The limitation of LG was that it could not provide a direct estimation of hazard ratios. By using the maximum likelihood methods with the Cox model, we reported the estimated hazard ratio of risk factors, especially for cancer subtypes and the positive MRD status, useful for comparison with other full cohort–based studies.

The advantage of Opt against SCCH becomes smaller when the event of interest is rarer. If one is willing to sacrifice some power advantage, SCCH can be a good choice in terms of its scalability, and flexibility of analyzing other outcomes.^19^ For complex second-phase designs where CoxW is not applicable, for example, when a region of the follow-up time is not sampled in the second phase, a computationally efficient strategy could be that we first use LG or TaoLG for initial scanning, then for those significant associations, we use TaoCox for consistent estimation of the effect sizes.

Odds ratio from the LG models and hazard ratio from the Cox models are distinct measures of effect sizes. Their interpretations are different. For time-to-event outcome, hazard ratio is a more natural measure of effect size. For example, the reported effect size using the odds ratio from the MP2PRT analysis using the SECC design depends on the specific definition of cases and controls, and cannot be directly compared with the hazard ratios from other full cohort–based analyses. However, the estimated hazard ratio using Tao's maximum likelihood with a Cox model from the MP2PRT analysis can be directly compared with other full cohort–based analysis, which is valuable for either validation in another cohort or meta-analysis purpose.

We note that the implementation of analysis methods may also contribute to the differences in computing time. Although LG and Cox have mature R packages and are well optimized, Tao's package for the nonparametric maximum likelihood method is more recent and likely has more room to improve. Therefore, computational time could be influenced more by software development and optimization than by the underlying theoretical methods.

WGS has the advantage of covering the whole genomes over other lower-cost genomic technologies, such as the targeted sequencing panels of candidate genes or regions, or polymerase chain reaction (PCR)–based method for specific DNA sequences. Therefore, WGS is the best option when comprehensive examination is needed between molecular biomarkers and the outcomes. Once the associations between the WGS-based molecular biomarkers and the outcome are discovered and confirmed, the lower-cost sequencing panels or PCR methods can be developed subsequently to obtain the biomarkers for a large-scale application.

Given the power and analysis advantages of combining both the first phase and second phase data, it is important to share both the inexpensive variables measured on all samples in the first phase and expensive variables in the second phase under proper data protection regulations. Several biobanks such as UK Biobank^20^ and All of Us^21^ are good examples in this regard where both medical and health-related variables for almost all recruited samples are shared, as well as the molecular data, although some are only available for a subset of the samples.

In this work, we focus on the MP2PRT study, which motivated our study design and evaluation work. The proportion of the relapse event for standard-risk B-ALL is about 10%, so the power difference between the SECC design and Opt is likely small. Our simulation shows that when the case is common, there will be a large power gain for Opt. Other high-prevalence diseases or conditions will fall into this scenario. However, further explorations of more specific settings reflecting the real diseases/conditions are needed to make sure our comparison results can be generalized to other diseases/conditions.

## Data Availability

A data sharing statement provided by the authors is available with this article at DOI https://doi.org/10.1200/CCI-24-00223.
